# Outcomes of Patients with Intestinal Failure after the Development and Implementation of a Multidisciplinary Team

**DOI:** 10.1155/2016/9132134

**Published:** 2016-05-19

**Authors:** Sabrina Furtado, Najma Ahmed, Sylviane Forget, Ana Sant'Anna

**Affiliations:** ^1^Department of Pediatrics, Montreal Children's Hospital, McGill University, 1001 Boulevard Decarie, Montreal, QC, Canada H4A 3J1; ^2^Department of Pediatrics, Division of Gastroenterology and Nutrition, Montreal Children's Hospital, McGill University, 1001 Boulevard Decarie, Montreal, QC, Canada H4A 3J1

## Abstract

*Aim*. A multidisciplinary team was created in our institution to manage patients with intestinal failure (INFANT: INtestinal Failure Advanced Nutrition Team). We aimed to evaluate the impact of the implementation of the team on the outcomes of this patient population.* Methods*. Retrospective chart review of patients with intestinal failure over a 6-year period was performed. Outcomes of patients followed up by INFANT (2010–2012) were compared to a historical cohort (2007–2009).* Results*. Twenty-eight patients with intestinal failure were followed up by INFANT while the historical cohort was formed by 27 patients. There was no difference between the groups regarding remaining length of small and large bowel, presence of ICV, or number of infants who reached full enteral feeds. Patients followed up by INFANT took longer to attain full enteral feeds and had longer duration of PN, probably reflecting more complex cases. Overall mortality (14.8%/7.1%) was lower than other centers, probably illustrating our population of “early” intestinal failure patients.* Conclusions*. Our data demonstrates that the creation and implementation of a multidisciplinary program in a tertiary center without an intestinal and liver transplant program can lead to improvement in many aspects of their care.

## 1. Introduction

Intestinal failure (IF) is a comprehensive term that describes a state of malabsorption in which the intestine is unable to maintain energy, fluid, electrolyte, or micronutrient needs, leading to inadequate growth and development [1, 2]. This can result from intestinal obstruction, dysmotility, surgical resection, or a congenital defect. The most common cause of IF is Short Bowel Syndrome (SBS) which is associated with a high incidence of morbidity and mortality [3, 4].

Large pediatric centers, with well-established intestinal transplant programs, have reported improvements in communication and coordination between services [5] and improved outcomes including decreased mortality [6, 7] by using a multidisciplinary approach for children with IF, which allows for better coordination of care [8–11]. There are only a few reports of those multidisciplinary groups and Pediatric Hospital that are not transplant centers in Canada [7, 12].

Our institution, the Montreal Children's Hospital (MCH), is a tertiary hospital that, although not performing liver/small bowel transplant, has a very complex population of patients. In December 2009, a multidisciplinary team (INFANT: INtestinal Failure and Advanced Nutrition Team) was developed at the MCH with the goal of improving the coordination of the complex care required by this population.

The aim of our study was to assess the impact of implementing our multidisciplinary team on the clinical outcomes of patients with IF diagnosed and followed up at our institution.

## 2. Methods

We undertook a retrospective review of the outcomes of intestinal failure patients at our center following the implementation of this multidisciplinary team and compared those to a matched cohort followed up prior to the creation of the team.

### 2.1. Intestinal Failure Team Development

The INFANT is composed of professionals from gastroenterology, neonatology, general surgery, nursing, nutrition, pharmacy, social work, and occupational therapy. The primary goal of this multidisciplinary team is to coordinate the highly complex care of patients diagnosed with intestinal failure. Patients are referred to INFANT when the treating neonatologist and surgeon expect intestinal failure to develop or when neonates already experience difficulty progressing enteral feeds.

The group meets monthly to establish guidelines and discuss aspects of the care of the inpatient population. Additional weekly rounds are performed by gastroenterologists and daily visits from the treating team (neonatology, surgery, or general pediatricians). Discharged patients are followed up weekly by a gastroenterologist and a nutritionist from INFANT until clinical condition is stable enough to allow longer intervals between follow-up visits.

Protocols such as ethanol lock therapy (ELT) for prevention of catheter related blood stream infections (CRBSI) and fish oil based emulsions to reverse/stabilize total parenteral nutrition (TPN) cholestasis were put in place by INFANT.

The ELT protocol was created with the purpose of providing guidelines for the safe administration of ELT for the prevention of CRBSI as well as providing teaching guidelines for nurses to educate and train families and caregivers in the safe administration of this therapy in the home setting. The ELT requires injection of each lumen of the central venous catheter (CVC) with 70% ethanol for a minimum of 4 hours and maximum of 24 hours at a frequency of three times per week. The following criteria had to be fulfilled in order for a patient to qualify for this therapy: diagnosis of intestinal failure, weight greater than 5 kg if patient has a single lumen central venous catheter (CVC) or greater than 9 kg if patient has a double lumen CVC or subcutaneous port, 3-month corrected age, PN cycled off for a minimum of 4 consecutive hours, history of two CRBSI within a defined period of time (6 months), serum ethanol level less than 2.5 mmol/L before initiation of therapy, patent CVC lumen before initiation of therapy, silicone-based CVC, and parent/caregiver consent to the use of ethanol locks.

The protocol of fish oil based emulsions to reverse/stabilize TPN cholestasis was created with the purpose of standardizing the use of fish oil based lipid emulsions at the Montreal Children's Hospital. Up until the creation of INFANT, patients diagnosed with TPN cholestasis were started on fish oil based lipid emulsions based on the decision of the treating neonatologist. The protocol established criteria for the use of fish oil emulsion, which consisted of serum direct bilirubin >50 *μ*mol/L and anticipated need for TPN for >4 weeks. Infants with liver disease secondary to cystic fibrosis, inborn errors of metabolism, and infectious hepatitis were excluded. Once consent was obtained from parents and the treatment was approved by Health Canada, a dose of 1 g/kg of fish oil based lipid emulsion was started in conjunction with 1 g/kg of conventional lipid emulsion. If there was no improvement within 4 weeks, conventional intralipid was discontinued. Fish oil based lipid emulsion was discontinued once the infant reached full enteral feeds, as well as discontinuation of parenteral nutrition.

Suggested approaches to the diagnosis and management of CRBSI and small bowel bacterial overgrowth (SBBO) were also created by INFANT and disseminated to the treating team at our institution.

Regarding diagnosis and initial management of CRBSI and management of the catheter, suggested approaches included the collection of central and peripheral blood cultures for aerobes, anaerobes, and fungi which should always be drawn in febrile or unwell TPN patients with CVC, regardless of the presence of a clinical focus. Initial antibiotic therapy should target at least coagulase negative staphylococcus, as well as gram negative rods. In the case of CRBSI involving fungi, the infected catheter should be removed within 24 hours. In the case of bacterial infections, catheter salvage measures should be undertaken including antibiotic-lock during periods of TPN.

Finally the suggested approach to the diagnosis and treatment of SBBO included sampling of small bowel fluid (if access is available) to obtain specific bacterial counts, with identification and sensitivities in order to guide therapy. If flora was unknown and symptoms were mild to moderate therapy was started with metronidazole 10 mg/kg/dose two to three times per day in a cycled manner, one week out of every two to four weeks. If it is clinically obvious that patient improved while on treatment but does not tolerate being off antibiotics, then alternating between two to three agents was advisable (metronidazole, gentamicin, amoxicillin-clavulanate, and cephalexin).

### 2.2. Patient Population

Patients diagnosed with SBS, from 3 years before to 3 years after the creation of the multidisciplinary team, were eligible for inclusion (December 1, 2006 to November 30, 2012).

We used the Canadian Association of Pediatric Surgeons (CAPS) definition of SBS, namely, the need for parenteral nutrition (PN) for more than 42 days after bowel resection or a residual small bowel length of less than 25% expected for gestational age [5].

A search was performed through our institution's medical records for patients born between December 1, 2006, and December 15, 2012, diagnosed with necrotizing enterocolitis (NEC), volvulus, gastroschisis, Hirschsprung's disease (HD), intestinal atresia, small bowel perforation, dysmotility, gastroparesis, gastric necrosis, or meconium ileus. The resulting group of 334 patients was then divided into two cohorts based on year of birth; the cut point corresponding to the date INFANT was created.

Of 182 patients born between December 1, 2006, and November 30, 2009, with one of the diagnoses listed above, 155 did not fulfill criteria for SBS, leaving 27 subjects to form the pre-INFANT cohort.

**Figure 1:**
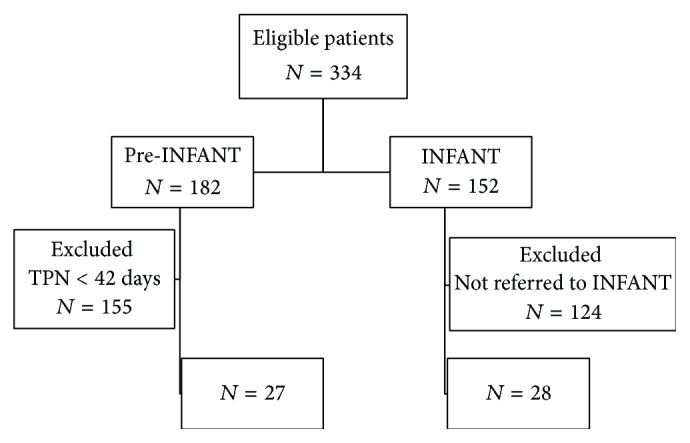
Flow chart of patient selection. INFANT = INtestinal Failure and Advanced Nutrition Team; TPN = total parenteral nutrition.

Of the 152 neonates born between December 1, 2009, and December 15, 2012, with one of the above-mentioned conditions, 28 were referred to INFANT due to a suspected or confirmed diagnosis of intestinal failure and constituted the INFANT cohort (Figure 1).

### 2.3. Data Collection

A retrospective chart review was conducted to obtain information on (a) demographics and clinical characteristics, (b) nutrition, and (c) morbidity and mortality, as described below.

The following information on patient demographics and clinical characteristics was abstracted: gestational age, birth weight, sex, primary diagnosis, etiology of SBS, type of surgery performed, presence of stoma, age at the time of surgery, small bowel and colon length remaining after surgery, presence of ileocecal valve (ICV), length of Neonatal Intensive Care Unit (NICU) stay and hospitalization, and length of follow-up by INFANT.

Nutritional data was obtained regarding duration of parenteral nutrition (PN) dependence, time to reach full enteral feeds if applicable, percentage of calories from PN if not fully enterally fed, home PN, gastrostomy tube insertion, and type of central venous line (CVL) if patient is on home PN.

Data on morbidity and mortality included number of septic episodes and etiology of sepsis, presence of cholestasis, maximum direct bilirubin, presence of liver failure, liver and bowel transplantation, and death and cause of death. The management strategies used to wean TPN in both groups were decided by the neonatologist and pediatric gastroenterologist in charge of that patient.

Operating room measurements of remaining small and large bowel were not available for all patients. In order to standardize the measurements, the percentages of small bowel and colon remaining after surgery were calculated by subtracting the length of intestine resected (data from pathology report available for all patients) from the average length of intestine for the patient according to the corrected gestational age at the time of the surgery [13, 14].

Length of follow-up by INFANT was calculated as having time 0 being the date when the gastroenterologist saw the patient for the first time, until the last outpatient visit (when follow-up ended before the completion of the study) or the date when data collection was completed (December 15, 2012) in the event that follow-up was still ongoing. Data on length of NICU and hospital stay, PN dependence, number and etiology of septic episodes, and cholestasis were obtained from electronic health record information.

Cholestasis was defined by a conjugated bilirubin level greater than 34 *μ*mol/L [14] and severe cholestasis was defined as conjugated bilirubin level greater than 50 *μ*mol/L. Liver failure was defined as a combination of a level of direct bilirubin exceeding 200 mmol/L for a minimum of 2 weeks, an international normalized ratio greater than 1.5, albumin level lower than 20 g/L, thrombocytopenia with counts lower than 100.000, signs of portal hypertension, or bridging fibrosis seen on liver biopsy [5].

### 2.4. Data Analysis and Statistics

All data was uploaded into the REDCap database system and exported into SPSS for analysis. Means of continuous variables were compared using *t*-test and proportions using Chi square, with a two-sided alpha value of 0.05. Patients from the pre-INFANT cohort were compared with patients from the INFANT group in all analyses.

## 3. Results

### 3.1. Demographics and Clinical Characteristics

The demographic and clinical characteristics of the pre-INFANT and INFANT groups were very similar (Table 1). The etiology of SBS was also similar in both groups except for the incidence of NEC that was higher in the pre-INFANT cohort (74.1% versus 39.3%; *P* = 0.009). Some patients had more than one etiology (i.e., gastroschisis and NEC).

The mean length of small bowel (115.6 cm versus 112.3 cm, *P* = 0.837) and colon (27.4 cm versus 18.3 cm, *P* = 0.245) remaining after surgery was similar in both groups. A number of patients had an initial stoma (37% versus 54%) and two or more surgeries (44% and 64%). No significant difference was seen with respect to preservation of ICV (70% versus 75%, *P* = 0.7), length of NICU stay (125.8 versus 115.8 days, *P* = 0.628), or length of hospitalization (160.5 versus 202.9 days, *P* = 0.107) between the pre-INFANT and INFANT cohorts.

### 3.2. Nutritional Outcomes and Surgical Interventions

The pre-INFANT and INFANT cohorts were comparable regarding number of patients that reached full enteral feeds (85.2% versus 85.7%, *P* = 0.956) although patients in the INFANT cohort took longer to attain full enteral feeds (100.8 versus 158.5 days, *P* = 0.014).

There was no significant difference in terms of number of patients on home PN (3.7% versus 7.1%, *P* = 0.574) but the duration of PN was longer in the INFANT cohort (107.9 versus 171.6 days, *P* = 0.006). Gastrostomy feeding tubes were more frequently used in the INFANT cohort (22.5% versus 46.4%, *P* = 0.059) although this did not reach statistical significance (Table 2).

### 3.3. Morbidity and Mortality

With regard to infectious complications there was no difference in the number of patients with at least one septic episode (18 versus 24, *P* = 0.096) as well as number of septic episodes per patient (2.83 in both groups). The presence of cholestasis was similar in both groups (85.2% versus 82.1%, *P* = 0.76) whereas peak direct bilirubin was significantly lower in the INFANT cohort (181.6 versus 116.6, *P* = 0.026). No patients were diagnosed with liver failure in either cohort. One patient in each group was assessed for bowel transplant at another center, but there were no patients transplanted at the time of data collection. There were no bowel lengthening procedures in any of the groups.

Overall mortality was lower after creation of the INFANT team (14.8% versus 7.1%, *P* = 0.362) but this was not statistically significant. The combined outcome of sepsis, severe cholestasis, and mortality did not differ between the groups (Table 3).

## 4. Discussion

The management of infants with SBS remains a major challenge. Great advancements have been made over the last few decades in the treatment of this condition. The prognosis of SBS has significantly changed with the development and subsequent refinement of parenteral nutrition, use of lipid reduction strategies and fish oil based lipid preparations, improvement of the care of central catheters, and intestinal lengthening procedures among other techniques [15, 16]. Despite these advancements, this patient population still suffers tremendous morbidity and mortality. Significant intellectual, emotional, and financial investments are still necessary [17].

Our study was performed with the goal of establishing whether the development and implementation of a multidisciplinary team, to follow up patients with intestinal failure, would improve their outcomes in a tertiary hospital that does not have a transplant program.

Analysis of patient demographics and clinical characteristics demonstrates that both groups had similar etiologies except for a higher number of patients with NEC in pre-INFANT cohort (74.1% versus 39.3%; *P* = 0.009). For reasons that are not clear, during the period of December 2006 to November 2009, there were twice as many patients diagnosed with NEC at the Montreal Children's Hospital when compared to the following 3 years.

There were a higher number of patients on home PN in the INFANT cohort (7.1% versus 3.7%; *P* = 0.574) as well as an overall longer duration of TPN (171.6 days versus 107.9 days, *P* = 0.006). One possible explanation could be that patients with more severe disease had improved survival after the establishment of our intestinal rehabilitation program, which can also provide intensive outpatient care. Since the number of patients in each group is relatively small, the survival of a small number of patients dependent on TPN could explain these findings.

Despite the longer duration on TPN, the INFANT cohort had a similar number of patients with at least one septic episode (85.7% versus 66.7%, *P* = 0.096) as well as number of septic episodes per patient (mean of 2.83 episodes per patient in both groups). This could possibly be attributed to improved infection control measures or reflect the fact that patients on home PN usually see their infection rates go down compared to when they are in hospital.

As it has been well described in the literature, the use of long term TPN may lead to parenteral nutrition associated liver disease (PNALD), a condition that if left untreated may progress to cirrhosis [2]. Those patients with liver failure may be left with small bowel and liver transplant as their only therapeutic option. In our study a comparable number of patients in both groups had cholestasis, but peak direct bilirubin was significantly lower in the INFANT cohort (116.6 ± 82.8 versus 181.6 ± 124.1; *P* = 0.026). Following the creation of INFANT, there were changes in management strategies including the early introduction of enteral feeds, standardization of the use of new omega-3 based lipid emulsions, cycling of TPN, and systematic use of prophylaxis for small bowel bacterial overgrowth, which could explain the improvement in peak bilirubin.

There was a trend towards increased use of gastrostomy tubes for enteral feeding in the INFANT group, which almost reached statistical significance (46.4% versus 22.2%, *P* = 0.059). Gastrostomy tubes are often preferred to nasogastric tube feeding in patients requiring long term enteral feeding access [18]. INFANT advocates for early insertion of gastrostomy tubes, a practice which was not as systematic at our institution prior to the establishment of this team [19].

Referral centers with established transplant programs report mortality rates around 30% [3, 5, 6, 20] prior to the establishment of multidisciplinary teams for the care of patients with intestinal failure. At our institution the mortality rate before INFANT was 14.8%, perhaps indicative of a less critically ill population. Despite dealing with a different population in terms of severity of disease (nontransplant center), the multidisciplinary approach to the care of patients with intestinal failure at our institution led to a 50% reduction in mortality (14.8% versus 7.1%, *P* = 0.362). This result would need to be confirmed in a larger number of patients, as our sample size was too small to reach statistical significance or yield precise point estimate of mortality. A recent systematic review of the impact of multidisciplinary intestinal rehabilitation programs on the outcome of pediatric patients with intestinal failure documented a reduction in septic episodes and an increase in overall patient survival [21].

This data demonstrates that the creation and implementation of a multidisciplinary program in a tertiary center without an intestinal and liver transplant program can lead to improvement in many aspects of their care.

We are currently collecting prospective data on the patients followed up by INFANT in order to evaluate the long term benefits of an intestinal failure team in our population and to use this information to optimize the care and long term outcomes of these complex patients.

## Supplementary Material

This is the data collection sheet.

## Figures and Tables

**Table 1:** Demographics and clinical characteristics of infants with SBS.

	Pre-INFANT (*n* = 27)	INFANT (*n* = 28)	*P*
Gestational age (weeks)	30.1 ± 4.9	31.1 ± 4.8	0.450
Birth weight (grams)	1473 ± 920	1736.8 ± 975	0.307
Male	14 (60)	11 (39)	0.349
CGA at first surgery (weeks)	35 ± 5.1 [21]	35.6 ± 8.7 [24]	0.768
*Etiology *			
Necrotizing enterocolitis	20 (74)	11 (39)	0.009
Intestinal atresia	3 (11)	5 (18)	0.478
Gastroschisis	2 (7)	4 (14)	0.413
Volvulus	1 (4)	4 (14)	0.172
Other	7 (26)	12 (43)	
*Outcomes after surgery*			
Length of small bowel (cm)	115.6 ± 24.6	112.3 ± 55	0.837
% of SB left	93.4 ± 10.4	84.5 ± 22	0.062
Length of colon (cm)	27.4 ± 6.2	18.3 ± 17.4	0.245
% of colon left	95.1 ± 11.9	83.4 ± 31.7	0.076
Presence of ICV	19 (70)	21 (75)	0.700
NICU stay (days)	125.8 ± 60.8	115.8 ± 87.6	0.628
Length of hospitalization (days)	160.5 ± 83.6	202.9 ± 106.6	0.107
Follow-up by INFANT (days)	NA	564.6 ± 286.1	NA

CGA = corrected gestational age; ICV = ileocecal valve; NICU = Neonatal Intensive Care Unit; SB = small bowel; INFANT = INtestinal Failure and Advanced Nutrition Team. Results are presented as mean ± SD or *n* (%); [ ] = *n* of patients for that specific variable.

**Table 2:** Nutritional outcomes and surgical interventions of patients with SBS.

	Pre-INFANT (*n* = 27)	INFANT (*n* = 28)	*P*
*Nutritional outcomes*			
Reached full enteral feeds [*n* (%)]	23 (85.2)	24 (85.7)	0.956
Days to reach full feeds (mean ± SD)	100.8 ± 68.6 [23]	158.5 ± 85.3 [24]	0.014
Home TPN [*n* (%)]	1 (3.7)	2 (7.1)	0.574
Duration of TPN (days; mean ± SD)	107.9 ± 68.9	171.6 ± 93.7	0.006
*Surgical interventions*			
Presence of CVL [*n* (%)]	1 (3.7)	2 (7.1)	0.574
Gastrostomy tube insertion [*n* (%)]	6 (22.2)	13 (46.4)	0.059

TPN = total parenteral nutrition; CVL = central venous line; results are presented as mean ± SD or *n* (%); [ ] = *n* of patients for that specific variable.

**Table 3:** Morbidity and mortality of infants with SBS.

	Pre-INFANT (*n* = 27)	INFANT (*n* = 28)	*P*
*Infectious complications*			
Patients with at least 1 septic episode [*n* (%)]	18 (66.7)	24 (85.7)	0.096
Number of septic episodes per patient (mean ± SD)	2.83 ± 2.66	2.83 ± 2.44	NS
*Liver disease*			
Presence of cholestasis [*n* (%)]	23 (85.2)	23 (82.1)	0.760
Peak direct bilirubin (mean ± SD)	181.6 ± 124.1	116.6 ± 82.8	0.026
Liver failure [*n* (%)]	0	0	NS
Assessed for transplant [*n* (%)]	1 (3.7)	1 (3.7)	NS
Transplanted [*n* (%)]	0	0	NS
*Mortality*			
Overall mortality [*n* (%)]	4 (14.8)	2 (7.1)	0.362
*Cause of death*	[4]	[2]	
Respiratory failure [*n* (%)]	2 (50)	1 (50)	NS
Cardiac arrest [*n* (%)]	1 (25)	0	NS
Cardiorespiratory failure [*n* (%)]	1 (25)	0	NS
Septic shock [*n* (%)]	0	1 (50)	NS
Liver failure [*n* (%)]	0	0	NS

INFANT = INtestinal Failure and Advanced Nutrition Team. Results are presented as mean ± SD or *n* (%); [ ] = *n* of patients for that specific variable.
