# Supplementary material for: Outcomes of Patients with Intestinal Failure after the Development and Implementation of a Multidisciplinary Team
Source: Can J Gastroenterol Hepatol. 2016 May 19;2016:9132134. doi: 10.1155/2016/9132134 (PMC4904660; doi:10.1155/2016/9132134)
Supplement: Supplementary file 1 — This is the data collection sheet. [file 9132134.f1.docx]

**Patient MRN: DOB:**

**Mother’s MRN: DOB:**

_________________________________________________________________________________________________

Top of Form

**INFANT team baby?
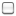
Yes
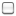
No**

**Patient’s Demographics/Clinical characteristics**

| Gestational age (weeks) |  |
| --- | --- |
| Birth weight (grams) |  |
| Sex (M/F) |  |
| Primary diagnosis |  |
| Etiology of IF |  |
| Type of surgery performed |  |
| Presence of stoma? |  |
| Age at time of surgery (weeks) |  |
| SB length (cm)/% of SB remaining |  |
| Colon length (cm)/% of colon remaining |  |
| Presence of IC valve |  |
| Length of NICU stay (days) |  |
| Length of hospitalization (days) |  |
| Length of follow up by INFANT – if applicable |  |

**Nutrition**

| Duration of PN dependance |  |
| --- | --- |
| Reached full enteral feeds? |  |
| Time (days) to reach full feeds |  |
| If not fully enterally fed, % of calories from PN |  |
| Home TPN? |  |
| G tube insertion? |  |
| Presence of CVL? Which type? |  |

**Morbidity/Mortality**

| # of septic episodes |  |
| --- | --- |
| Etiology of sepsis |  |
| Cholestasis? |  |
| Max Direct bili |  |
| Liver failure? Days to liver failure? |  |
| Assessed for transplant? |  |
| Listed for transplant? |  |
| Transplanted? |  |
| Death? Which cause? Liver failure? |  |

Bottom of Form
